# How to deal with dialysis catheters in the ICU setting

**DOI:** 10.1186/2110-5820-2-48

**Published:** 2012-11-23

**Authors:** Natacha Mrozek, Alexandre Lautrette, Jean-François Timsit, Bertrand Souweine

**Affiliations:** 1 RÃ©animation mÃ©dicale, HÃ´pital Gabriel Montpied CHU-Clermont-Ferrand, Clermont Ferrand, 63000, France; 2 UMR CNRS 6023, Laboratoire Microorganismes: Génome et Environnement, Clermont UniversitÃ©, Université d'Auvergne, Clermont Ferrand, 63000, France; 3 Medical Polyvalent Intensive Care Unit, University Joseph Fourier, Albert Michallon Hospital, BP 217, Grenoble Cedex 9, 38043, France; 4 University Joseph Fourier, EA U823, Albert Bonniot Institute, La Tronche Cedex, 38706, France

**Keywords:** Dialysis catheter, Intensive care unit, Catheter dysfunction, Catheter infection

## Abstract

Acute kidney insufficiency (AKI) occurs frequently in intensive care units (ICU). In the management of vascular access for renal replacement therapy (RRT), several factors need to be taken into consideration to achieve an optimal RRT dose and to limit complications. In the medium and long term, some individuals may become chronic dialysis patients and so preserving the vascular network is of major importance. Few studies have focused on the use of dialysis catheters (DC) in ICUs, and clinical practice is driven by the knowledge and management of long-term dialysis catheter in chronic dialysis patients and of central venous catheter in ICU patients. This review describes the appropriate use and management of DCs required to obtain an accurate RRT dose and to reduce mechanical and infectious complications in the ICU setting. To deliver the best RRT dose, the length and diameter of the catheter need to be sufficient. In patients on intermittent hemodialysis, the right internal jugular insertion is associated with a higher delivered dialysis dose if the prescribed extracorporeal blood flow is higher than 200 ml/min. To prevent DC colonization, the physician has to be vigilant for the jugular position when BMI < 24 and the femoral position when BMI > 28. Subclavian sites should be excluded. Ultrasound guidance should be used especially in jugular sites. Antibiotic-impregnated dialysis catheters and antibiotic locks are not recommended in routine practice. The efficacy of ethanol and citrate locks has yet to be demonstrated. Hygiene procedures must be respected during DC insertion and manipulation.

## Review

### Introduction

Acute kidney insufficiency (AKI) [1] requiring renal replacement therapy (RRT) occurs in approximately 4% of critically ill patients [2] and is associated with a mortality rate ranging between 38% and 82% [3]. Whereas vascular access is necessary for extracorporeal blood circulation during RRT, measures should be taken to preserve the vascular network, due to the higher risk of long-term chronic renal disease in these patients [4]. In critically ill patients, veno-venous access via dialysis catheters (DCs) rather than arteriovenous access is recommended [5]. Most information and recommendations on temporary DC insertion and management are based on data concerning the use of long-term DCs in patients with end-stage renal disease (ESRD) or that of central venous catheters (CVCs) in the intensive care unit (ICU). Whether such data may be extrapolated to DCs in critically ill patients is questionable, because critically ill patients differ widely from ESRD patients, DCs differ widely from CVCs with regard to manipulation and management, and RRT techniques differ widely between the dialysis unit and the ICU.

During the past decade, several reports have been published on the epidemiology of DC-related complications in ICU patients [6-15]. This review looks at the basic characteristics of dysfunction, thrombosis, and infections encountered with DC use in adult critically ill patients.

### DC dysfunction and thrombosis

#### DC dysfunction

Blood flow (*Q*_B_) through the DC is a major determinant of an optimal RRT dose [16]. The impact of DC on the actual delivered dose is greater in intermittent than in continuous RRT techniques, because the latter use lower *Q*_B_ and therefore are less dependent on DC performance. In chronic hemodialysis patients, DC dysfunction is defined as the failure to attain a sufficient extracorporeal blood flow of ≥300 mL/minute with a prepump arterial pressure below −250 mmHg [17]. In critically ill patients, there are numerous definitions of DC dysfunction, most of which apply to patients on intermittent hemodialysis [18-20]. In these studies, DC dysfunction is defined as the inability to attain and maintain blood flows of at least 150 ml/min [18], a low blood flow (<200 mL/min) incapable of providing adequate dialysis [19], and *Q*_B_ reduction >20% despite attempts to restore patency [20]. In a recent study, DC dysfunction in patients on continuous RRT was defined by the necessity to replace the DC because of an inability to attain an adequate *Q*_B_ through the DC [11]. In clinical practice, temporary DC dysfunction can be defined as the failure to attain and maintain *Q*_B_ through the DC sufficient for the administration of an adequate RRT dose. DC dysfunction can be diagnosed as frequent arterial and venous pressure alarms, which reveal a high negative outflow pressure and an increase in venous pressure. DC dysfunction can be indicated by low values of urea reduction in patients on continuous or intermittent RRT techniques [21] or by low Kt/V values in patients on intermittent hemodialysis [22]. In the latter, the delivered RRT dose can be easily estimated by ionic dialysance measurement [23]. The RRT dose should be prescribed before each session of RRT and the actual delivered dose regularly assessed. In the VA/NIH study, there were no differences in outcome between patients receiving a weekly 3.9 Kt/V and a weekly 7.8 Kt/V [24]. It is therefore recommended to deliver a Kt/V of 3.9 per week [5]. In continuous RRT, the VA/NIH study and the RENAL study showed no differences in mortality between doses of 20 ml/kg/h and 35 ml/kg/h nor between 25 ml/kg/h and 40 ml/kg/h [24,25]. In continuous RRT, it is recommended to deliver an effluent volume of 20–25 ml/kg/h [5]. Immediate or early DC dysfunction usually results from improper catheter placement, such as insertion in the wrong vessel, malposition of the catheter tip (sucking the wall of the vein), and kinking of the catheter, and strictures caused by ligatures or fascia. Inadequate *Q*_B_ that occurs late after DC insertion more often is caused by thrombotic problems, either intrinsic (partial or total obstructive thrombosis of the catheter lumen) or extrinsic (thrombosis or stenosis of the cannulated vein) catheter thrombosis [4].

In the literature, the rates of DC dysfunction differ because of differences in study design and the definitions of dysfunction. In one survey of 73 DCs, the DC dysfunction rate was 31.5% [18]. In the Cathedia study, when only the first DC placement was assessed, the rate of DC dysfunction was 10.7% (74/690) [11] but increased to 24.2% (65/268) in the 134 patients with two consecutive DCs inserted at an alternative site. The rate of DC dysfunction increases with DC placement duration and is higher beyond the first week of DC insertion [18]. However, most DC dysfunctions occur within the first 10 days of DC placement [11].

Preventive strategies of DC dysfunction include ultrasound guidance for a higher chance of DC placement [5], checking for adequate flow immediately after placement, flushing each DC lumen with saline at the beginning and end of each RRT session, anticoagulant locking followed by careful clamp closing, and checking the correct position with x-rays. In a recent, prospective, randomized, monocentric study involving 78 patients in a surgical ICU, citrate catheter lock solution, by comparison with serum saline lock solution, reduced DC dysfunction and extended DC life span [20]. When dealing with DC dysfunction, classical measures to improve *Q*_B_ include repositioning the patient, flushing the DC with saline, rotating the DC, and reversing the lines. In hemodynamically stable patients with low KT/V values and persistent low *Q*_B_ (*Q*_B_ <200 ml/min) in the extracorporeal circuit despite implementation of the above measures, the question of DC replacement should be addressed.

#### Materials of the catheter

Temporary DCs are usually made of thermoplastic elastomers that are rigid at room temperature to facilitate insertion and that soften at body temperature to minimize vessel lesions. Silicon DCs are less thrombogenic than polyurethane DCs [26,27]. For an equal outer diameter, polyurethane DCs have thinner walls than silicone DCs and provide less resistance to *Q*_B_. Catheter integrity may be altered by antiseptic solutions. Polyvidone iodine has no impact on polyurethane but induces a structural degradation of silicone. Ethanol exposure has only a marginal impact on silicone DC integrity; in contrast, prolonged exposure of polyurethane DCs to concentrated ethanol should be avoided [28].

Because DCs are thrombogenic, manufacturers have designed DCs coated with heparin. In chronic dialysis patients, heparin-coated catheters are safe but do not reduce catheter dysfunction [29-31]. There are no published, clinical studies in adult critically ill patients that compare DC dysfunction rates for heparin-coated and noncoated DCs.

#### Tunneled DCs

One prospective study compared tunneled and non-tunneled DCs inserted at the femoral site in 30 critically ill patients and showed that in patients with tunneled DCs, DC dysfunction occurred less frequently, and the delivered RRT dose and catheter duration were higher [9]. However, the insertion procedure of tunneled DC took longer and resulted in more femoral hematomas [9].

#### DC lumens

Venovenous catheterization can be performed with two single-lumen DCs either placed side by side in the same vein or in two different veins, and with a dual-lumen DC or a triple-lumen DC that has a third lumen for fluid and medication administration or blood sampling. In a retrospective study performed in critically ill patients on intermittent hemodialysis involving 534 DCs, the incidence rate of thrombotic complications was significantly higher in dual-lumen than in single-lumen DCs [19]. In most cases, the higher incidence rate of dual-lumen thrombotic complications was due to thrombosis of the arterial line.

Several internal configuration lumens are available for dual-lumen DCs, including double D, double C, coaxial, and shotgun lumens. Semirigid polyurethane dual-lumen DCs with shotgun lumens and no side holes are the most popular devices because of their ease of insertion and good flow characteristics [32].

#### Distal ports of DCs, positioning and length of the catheter

The adequacy of the delivered RRT dose decreases proportionally with the increase in blood recirculation. Blood recirculation occurs when dialyzed blood returning through the venous DC lumen reenters the extracorporeal circuit through the arterial lumen, rather than returning to the systemic circulation. Several mechanisms contribute to blood recirculation, such as Foucault current due to turbulent flow and catheter tip clotting, obstruction flow in the vein, reversal of flow in the central venous system during the atrial systole (normal pulsatile flow, tricuspid regurgitation), sheathing of the catheter with fibrin, which can create a pathway from the outflow to the inflow port, positive pressure ventilation, and reversal of the outflow and inflow lines. Blood recirculation is minimized when the venous outlet is positioned in a large central vein with a high blood flow rate and therefore depends on the length of DCs. Because blood recirculation depends mainly on the ratio of the flow in the catheter and in the vessel in which it is located, its impact on the delivered RRT dose is lesser in continuous RRT, which uses lower *Q*_B_ than intermittent hemodialysis.

The tips of DCs inserted in the upper body have to be placed close to the right atrium to provide a better *Q*_B_. Because polyurethane DCs are made of rigid plastic, they must not be extended beyond the superior vena cava to avoid right atrial trauma. For internal jugular access, both 15–20-cm silicone DCs targeting the superior vena cava and 20–24-cm soft silicone DCs targeting the right atrium appeared to be safe in critically ill patients. However, the silicone DCs with right atrial placement also improved dialyzer life span [33]. For femoral access, DC tips should be placed in the inferior vena cava to minimize blood recirculation because the lower flow in the smaller veins can dip below the pumped flow. Femoral catheters <20 cm have significantly greater blood recirculation than those >20 cm [34]. In critically ill patients, DCs with a minimal length of 24 cm could possibly be used, because compared with 24-cm femoral DC 20-cm femoral DCs are independently associated with a diminished urea reduction ratio [11].

#### Insertion site

Because end-stage renal disease may complicate AKI, subclavian access for DC placement should be proposed as a last resort for fear of vein stenosis, which compromises permanent access in critically ill patients [5]. Prospective surveys of DCs in critically ill patients suggest that internal jugular access might be preferable to femoral access to minimize DC dysfunction [35] and blood recirculation [36], and to improve RRT provision [37]. The results of recent studies contrast with these data. In the Cathedia study, which included 750 patients from 12 different ICUs, the rate of DC dysfunction of the first RRT vascular access was similar in the femoral (36/348, 10.3%) and the internal jugular routes (38/342, 11.1%), and the time to DC dysfunction did not differ between the two access sites [11]. These results were confirmed in a subgroup analysis performed in 134 patients who received two successive DCs at two different insertion sites (either femoral or internal jugular) and that showed a rate of DC dysfunction of 22.4% with femoral access and of 26.1% with internal jugular access [13]. However, when the sides of site insertion are compared, there is a trend to a lower rate of DC dysfunction in the right internal jugular site (6.6%) than in the femoral sites (10.3%). DC dysfunctions are more frequently observed at the left internal jugular site (19.5%) than at the right internal jugular and femoral sites [11].

Among patients starting with intermittent hemodialysis, there is no difference in the RRT dose as evaluated by the urea reduction ratio (URR), between femoral and internal jugular accesses [11]. Independent factors of higher URR in multivariate analysis were female gender, lower weight, higher predialysis urea value, and longer session duration. A reduced URR was associated with 20-cm DCs by comparison with 24-cm DCs at the femoral site. For a *Q*_B_ < 200 ml, there was no difference between the femoral and the internal jugular routes. In contrast, *Q*_B_ > 200 ml was associated with a higher URR when DCs were inserted at the internal jugular site [11].

In conclusion, when considering DC dysfunctions and the delivery RRT dose, the internal jugular and femoral routes are equivalent. Because right internal jugular access offers the straightest route to the superior vena cava and allows higher *Q*_B_, it should be used as the first choice for DC placement in patients treated with intermittent hemodialysis if the prescribed *Q*_B_ is higher than 200 ml/min. Ultrasonography guidance, which reduces the risk of catheter placement failure [38-40] and of insertion-related complications, is recommended for temporary DC placement in AKI patients [5], particularly for internal jugular access, which is more frequently associated with life-threatening catheter-insertion complications than femoral access [10].

#### DC-related thrombosis

##### Intraluminal DC thrombosis

Strategies for the prevention of intraluminal thrombosis include forcible flushing with saline of both DC lumens to clear them of blood at the beginning and at the end of each RRT session, anticoagulant interdialytic locking, and careful closing of the clamp on the catheter after interdialytic lock instillation. Concentrated heparin is the most popular interdialytic anticoagulant solution used for DC locking, even though no clinical study in critically ill patients has assessed its efficacy and safety. A recent, prospective, randomized, monocentric study of 78 patients in a surgical ICU suggests that 46.7% sodium citrate locks reduce DC dysfunction rates and increase DC life span compared with serum saline locks [20]. When thrombosis is limited to the DC lumen, DC locking with fibrinolytic agents, such as urokinase and alteplase, is effective in restoring DC patency in chronic dialysis patients [17]. Whether this intervention is safe in critically ill patients remains unknown and therefore the use of fibrinolytic locks cannot yet be recommended.

##### DC-associated vascular thrombosis

Very few data are available for DC-associated vascular thrombosis in critically ill patients. In the Cathedia study, the rate of symptomatic deep venous thrombosis was 0.5% (4/736) and did not differ between internal jugular and femoral DC placement. In the two centers of the study in which the presence of DC-associated thrombotic complications was systematically assessed by ultrasonography, the rate of thrombosis was 16.5% (25/151), with a trend to a higher rate in the internal jugular arm (22.7%) than in the femoral arm (10.5%) [10]. There are no published reports on the management of DC thrombosis. However, when dealing with asymptomatic or symptomatic DC-associated vascular thrombosis in the ICU setting DC removal is mandatory.

#### Guidewire exchange

In chronic dialysis patients, it is recommended to use a guidewire for replacement of a malpositioned catheter inducing DC dysfunction [17]. Guidewire exchange in the event of DC dysfunction often is performed in ICU patients, but its efficacy and adequacy have not been assessed. Further studies are needed in the ICU setting to determine the impact of this strategy.

#### Catheter management

Catheter dysfunction rates differ between ICUs [11], suggesting that procedures, teaching and training of staff and physicians concerning DC placement, and maintenance play a major role in RRT management and provision [41]. Procedures to prevent catheter dysfunction are given in Table 1.

**Table 1:** Prevention and management of catheter dysfunction

**Choice of the dialysis catheters**	
Materials	Silicone or polyurethane catheter
	Heparin coated catheters are not recommended
Diameters	12- to 16-French (4–5 mm)
Length	For the upper sites: at least 15 cm to obtain right atrium placement for soft DC, superior vena cava for rigid DC
	For the lower sites: probably at least 24 cm
Lumens	Dual lumen catheter
	Two single-lumen catheters less easy to place but at least as accurate as dual lumen catheters
Tunnelization	Lower rate of DC dysfunctions but placement more difficult
**Choice of the insertion site**	
	Femoral and right jugular sites better than left jugular site
	Right internal jugular site should be preferred in intermittent hemodialysis if *Q*_B_ has to be higher than 200 ml/min
	Subclavian sites to be avoided
	Ultrasound guidance especially for jugular sites
	Preserve vascular network
**Positioning of the catheter**	
Upper sites	Tips of the catheter placed next to the right atrium in the superior vena cava
	Check chest radiography
Lower sites	Tips of the catheter placed in the inferior vena cava
**During renal replacement therapy (RRT)**	
Flush	Use saline solution flushes before and after every RRT session
Pressure	Check pressure greater than −250 mmHg on the inflow site
	Check pressure <250 mmHg on the outflow site
Lock	Anticoagulant lock, i.e., heparin after every RRT
Clamp	Careful clamp closing after every RRT
**In case of dysfunction**	
Patient	Try to change patient position
Flush	Try to flush catheter lumens with saline solutions
Catheter	Try to rotate the catheter
Lumens	Try to reverse catheter lumens. Prolonged port reversal not recommended due to recirculation which compromises efficacy
Locks	Fibrinolytic locks are not evaluated and are not yet recommended
Dose of RRT	Check previous KT/V in case of intermittent hemodialysis session and consider catheter replacement
**Education of the team**	

### DC-related infections

Since a review on the prevention of catheter related infection in ICU was recently published in this journal [42], this chapter will focus on the epidemiologic particularities, and preventive measures specifically tested in ICU patients with DCs. The definitions of DC infections are classically extrapolated from the definitions used for CVC infections [42]. DC colonization is defined by a positive semiquantitative [43] or quantitative [44,45] DC tip culture. DC-related clinical sepsis is defined by DC colonization and resolution of clinical sepsis after DC removal in the absence of any other infectious site or initiation of new antibiotic therapy or both. Exit site infection is diagnosed by the presence of pus at the insertion site. DC-related bloodstream infection is defined as the isolation of the same phenotypic microorganism from peripheral blood culture and from DC tip culture or by a differential time to positivity of at least 120 minutes between blood cultures centrally drawn from DC and from a peripheral vein, when there is no other overt source for the bacteremia except the DC. The main trials studying DC infection in ICU are given in Table 2.

**Table 2:** Characteristics of the main trials studying dialysis catheter infection in ICU

**Author**	**Date**	**Study design**	**No. of patients**	**Catheters number**	**Site of insertion**	**Catheter tip culture**	**Catheter-related infection definition**	**Colonization (/1,000 c.d.)**	**Catheter-related infection (/1,000 c.d.)**	**TC (days, mean ± SD)**
Souweine^a^	1995-1996	Prospective, open, monocentric	170	151	Femoral and jugular	Simplified Brun Buisson	**CRBSI:** catheter colonization and blood culture positive for the same organism; site **infection**: presence of pus at the insertion site.	24.2	1.5	6.8 ± 6
Wester^a^	1997-1998	Prospective, open, monocentric, CAVHDF, ICU	43	139	Axillary arteries, femoral veins and arteries, subclavian veins	Semiquantitative culture: >15 CFU; quantitative culture: >10^3 CFU	**Exit site infection:** erythema, tenderness, induration, or purulence within 2 cm of the skin at the exit site of the catheter; **CRBSI**: Same organism isolated from a culture of the catheter and from the blood with clinical symptoms of infection; in the absence of laboratory confirmation, defervescence after removal of a catheter may be considered indirect evidence of CRBSI.	46.8% vs. 39.1%	2.2%	4.2 ± 2 vs. 7.3 ± 4.5
Harb^a^	1998-1999	prospective, open, monocentric, ICU	47	79	Femoral, subclavian, and jugular	Simplified Brun Buisson	**Infected catheter:** positive catheter tip culture with clinical signs of sepsis resolving within 48 hours after catheter removal; **CRBSI**: same microorganism isolated from the catheter tip culture and from cultured peripheral blood culture drawn during catheter placement or within the 24 hours following removal of the catheter. Differential time of positivity >2 hours.	5.4 (3.7%)	1.8 (1.2%)	6.9 ± 5.5
Chatzinikalaou^b^	2000-2002	prospective, randomized, monocentric, antibiotic coated dialysis catheters, 82% ICU	130	130: 66 antibiotic coated vs. 64 non-coated catheters	Femoral	Sherertz	fever (>38°C), chills, hypotension, skin organisms cultured from at least one blood cultures from a peripheral vein that was not related to infection of another site, and antimicrobial therapy; same organism isolated from peripheral blood culture and from DC tip culture (>1,000 CFU); presence of a positive quantitative catheter culture in a patient with clinical signs of sepsis that disappeared within 48 hours after catheter removal.	22% of all catheters (20% of antibiotics coated catheters vs. 25% of uncoated catheters)	14.3 (11% of uncoated catheter)	8 ± 6
Souweine^a^	2001-2004	prospective, open, monocentric	99	130	Femoral and jugular	Simplified Brun Buisson	**CRBSI**: isolation of the same phenotypic microorganism from both peripheral-blood culture and catheter-tip culture growing greater than 10^3 CFU/mL when there was no other source for bacteremia.	9.1	0	6.7 ± 4
Schönenberg^a^	2003-2007	prospective, open, monocentric	173	173	Subclavian, jugular, and femoral	NR	**CRBSI**: criteria for laboratory diagnosis of infection and clinical signs of sepsis. Laboratory diagnosis of infection is defined as a positive blood culture with a strain not descending from a different site of infection.	NR	3.8	9.2
Klouche^b^	2004-2005	prospective, monocentric, randomized, ICU	30	30: 15 tunneled vs. 15 non- tunneled catheters	Femoral	NR	Association of fever or chills or an overtly purulent exit site with a positive catheter clot or catheter culture result	NR	6.7%	13.5 ± 9.2 (tunneled) vs. 5.6 ± 3.4 (non-tunneled)
Parienti^b^	2004-2007	prospective, multicentric, randomized, few coated catheter (21%), ICU	637	637: 366 jugular vs. 370 femoral catheters	Femoral and jugular	Simplified Brun Buisson	catheter tip colonization plus at least one peripheral blood culture yielding the same species with the same antimicrobial susceptibility as the catheter tip within 48 hours of catheter removal, with no other apparent source of sepsis	40.8 (25.9%, femoral catheter) vs. 35.7 (24.9%, jugular catheter)	1.5 (0.5%, femoral catheter) vs. 2.3 (0.5%, jugular catheter)	4.9 ± 2
Parienti^b^	2004-2007	prospective, multicentric, randomized, few coated catheter (21%), ICU	637	637: 470 intermittent RRT vs. 266 continuous RRT	Femoral and jugular	Simplified Brun Buisson	catheter tip colonization plus at least one peripheral blood culture yielding the same species with the same antimicrobial susceptibility as the catheter tip within 48 hours of catheter removal, with no other apparent source of sepsis	38.9 (25.4%) [42.7 (intermittent hemodialysis) vs. 27.7 (continuous renal replacement therapy)]	1.9 (1.3%) [2.6 (intermittent hemodialysis) vs. 1.2 (continuous renal replacement therapy)]	6.3 (6.2) vs. 6.6 (6)
Dugué^b^	2004-2007	prospective, multicentric, randomized, few coated catheter (21%), ICU	134	268: 57 femoral then jugular vs. 77 jugular then femoral catheter	femoral and jugular	simplified Brun Buisson	NR	25,4% (femoral catheter) vs. 26,9%(jugular catheter)	NR	7.9 (5.6)
Skofic^a^	2004-2008	retrospective, monocentric, prospectively data collection	290	534	femoral, subclavian, and jugular	NR	**exit site infection**: local inflammation with purulent discharge and positive exit site culture; **suspected CRBSI**: proven systemic infection without any other recognized source of infection; **confirmed CRBSI**: at least one positive blood culture from a peripheral vein along with at least one positive blood culture from the catheter or positive catheter tip culture with an identical microorganism; **possible CRBSI**: at least one positive microbiological culture, good clinical response to catheter removal and antibiotic therapy, but lacking all criteria for confirmed CRBSI.	NR	4.6 (5.2%)	11
Hermite^b^	2009-2010	prospective, monocentric, randomized, ICU	78	135: 77 saline vs. 58 citrate lock	femoral and jugular	NR	**CRBSI**: fever (>38°C) with concordant positive blood cultures drawn from the catheter and a peripheral vein or a peripheral blood culture and a concordant exit site culture; **probable CRBSI**: fever with one positive blood culture, in the absence of any other clinically identifiable source of infection other than the catheter.	NR	30 (saline lock) vs. 24 (citrate lock)	6 [3-10] saline lock group vs. 12 [8-17] citrate lock group

*TC* time of catheterization; *ICU* intensive care unit; *CAVHDF* Continuous arteriovenous hemodiafiltration; *CRBSI* Catheter-related bloodstream infection; *CFU* Colony-forming unit; *NR* not related; Simplified Brun Buisson and Sherertz as previously described [44,45]^a^Observational descriptive studies; ^b^comparison studies.

#### Mechanisms and incidence of DC-related infections

In short-term catheterization, the exoluminal route is considered the main mechanism of catheter colonization. Frequent manipulations during DC use may predispose the endoluminal route to DC colonization. However, the etiologic organisms of short-term DC and central venous catheters are similar whatever the insertion site. This suggests that the route of colonization may not differ between these two types of catheters [6,10].

#### Measures for preventing DC infections

Procedures to prevent DC infections are summarized in Table 3.

**Table 3:** Prevention of dialysis catheter infection

**Choice of the dialysis catheter**	
Lumens	No difference between dual lumen catheter and two single lumen catheters placed side by side in terms of infection
Tunnelization	Not recommended for initiating RRT
Antimicrobial-coated catheters	Use not currently recommended and should be limited to units with high rates of DC infections despite implementation of adequate preventive strategies
**Choice of the insertion site**	
	No difference between femoral or jugular sites in term of infection.
	Physicians should be vigilant with femoral site in case of high body mass index, and with internal jugular site in case of low body mass index
**Insertion procedures**	
Hygiene procedure	Surgical hand disinfection
Depilation	Wear a long-sleeved sterile gown, sterile gloves, and cap
	Use a large sterile drape
	If hairs disturb vascular puncture or dressing occlusion
Skin preparation	>0.5% alcoholic chlorhexidine or alcoholic povidone iodine
Antibiotic prophylaxis Ultrasound guidance	Not recommended. May be proposed for internal jugular DC placement
**During RRT**	
Hygiene procedure	Use strict aseptic conditions for every DC manipulation
Dressing	Limit manipulation
	Avoid use of dialysis catheter for perfusion or blood samples, except in case of life threatening emergency
	Semipermeable transparent polyurethane dressing, sterile gauze
Antimicrobial lock solutions	Before applying a new dressing, clean skin with antiseptic solution, 0.5% alcoholic chlorhexidine or alcoholic povidone iodine
	Change in case of disruption or soiled dressing
	Change dressings at every dialysis
	Not recommended for prevention
Local ointments	Not recommended for ICU dialysis catheter
Catheter	Catheter replacement not scheduled
	Limit indwelling time and remove as soon as unnecessary

##### Hygiene precautions insertion technique and general policy

No specific study has been performed on this topic for DC management in the ICU setting, and therefore most available data are derived from studies on central venous catheters. The main findings were published recently in this journal [42]. In an observational study performed in critically ill patients, there was no difference in the rates of catheter colonizations between central venous and DCs when similar infection control measures were used for insertion and maintenance [8]. Thus, as recommended for central venous catheters [46] and dialysis catheters in end-stage renal disease patients [47], DCs in ICU patients should be inserted using maximal sterile barrier precautions and be manipulated under strict aseptic conditions. Skin antisepsis should be performed with >0.5% chlorhexidine preparation with alcohol or alcoholic-povidone iodine [46]. One study demonstrated that ultrasound guidance before insertion in the internal jugular vein reduces the rate of central venous catheter-related bacteremia [48]. No study has specifically assessed whether ultrasound guidance on DC insertion reduces DC infections in critically ill patients. Continuous training and competence testing of medical and nursing staff along with assessment of compliance with preventive measures and of feedback to health care workers should be included in preventive strategies of DC infections [47].

##### Antimicrobial coating and catheter selection

Regarding the risk of DC infections in relation to the number of DC lumens, only one study, which was retrospective, has focused on this topic and reported no differences in DC infections between patients with two single-lumen DCs and those with double lumen DCs [19]. One randomized, control study evaluated the impact of antimicrobial-coated DCs in critically ill patients [49]. It included both critically and non-critically ill patients with femoral non-tunneled DCs. The rate of DC-related infections was significantly lower in patients with rifampin-coated DCs compared with standard DCs. In the group with standard DCs, of the seven patients with infection, only one developed a DC-related bloodstream infection as defined by both blood cultures and catheter cultures positive for the same microorganism. In addition, there is a potential risk for emergence of antibiotic resistance when using antibiotic-coated DCs. As recommended for central venous catheters [46], the use of antimicrobial-coated DCs should be limited to units with high rates of DC infections despite the implementation of adequate preventive strategies. Whether tunneled DCs may be used for preventing DC infections in the ICU setting requires additional investigation, because only one small study, involving 30 patients, has addressed this issue and did not provide conclusive results [9]. Initiating RRT via tunneled DC is not yet recommended [5].

##### Site of insertion

By contrast with long-term DCs, there are no differences in short-term, DC-related infections between femoral and internal jugular accesses in critically ill patients [6,8,10]. However, when patients were stratified according to body mass index, the rate of DC-related colonization was higher at the femoral site for patients with a body mass index >28.4 and higher at the internal jugular site for patients with a body mass index <24.2 [10]. Because a relationship may exist between risk of colonization and the risk of infection [50], clinicians should be vigilant when the femoral site is used in patients with high body mass index, and the internal jugular site in patients with low body mass index.

##### Exit site care

Because of the lack of documented evidence, local antiseptic ointment and antiseptic- impregnated dressing cannot be recommended for DC management in critically ill patients.

##### Systematic/prophylactic changing and catheter indwelling duration

The rate of DC-related colonization/infection increases with DC duration, and therefore, DCs must be removed as soon as they are no longer needed [6,7,12]. The risk of DC colonization increases after 10 days among patients starting continuous RRT. In contrast, the duration of catheterization does not influence the daily hazard rate of DC tip colonization among patients starting with intermittent hemodialysis [12]. Additional data are needed to recommend systematic DC replacement every 10 days in patients treated with continuous renal replacement therapy. More information are needed before routine scheduled changes of DC can be recommended. DCs should be removed as soon as no longer needed.

##### Effects of RRT on catheter-related infection

Continuous renal replacement therapy may limit the number of catheter manipulations but can increase the risk of hypothermia compared with intermittent hemodialysis. A subgroup analysis of the Cathedia cohort shows that there are no differences between these two types of RRT in terms of infection of vascular access [12].

##### Antimicrobial/other DC solution lock

Antimicrobial lock consists in instilling and maintaining an antimicrobial solution in the catheter lumen to limit endoluminal biofilm formation and subsequent catheter-related bloodstream infection. Heparin is classically used as an interdialytic lock solution in chronic dialysis patients. Whether heparin lock solutions have antimicrobial activity is debated, because experimental studies assessing the antibiofilm properties of heparin have yielded conflicting results [51-53].

Antibiotic-based lock solutions are effective in preventing DC-related bloodstream infections in end-stage renal disease patients with tunneled and cuffed CVCs [54-57]. However, a number of issues remains concerning the use of the antimicrobial lock method for the prevention of DC-related infections, including the risk of developing bacterial resistance [58], and systemic toxicity due to leakage of these solutions [59]. Thus, using antibiotic locks for the prevention of catheter-related infections of non-tunneled DCs is discouraged [5].

Other promising antimicrobial lock solutions have been studied in chronic hemodialysis patients. Taurolidine is an antibiotic with anti-lipopolysaccharide properties that is not used to treat systemic infections. A recent study failed to demonstrate that 1.35% taurolidine 4% citrate lock solution reduces DC-related bacteremia compared with heparin [60]. The development of antibiotic-resistant organisms may be prevented in part by using chemical-based antimicrobial solutions, such as concentrated citrate and methyl blue paraben. Concentrated citrate lock solution has been reported to reduce DC-related bacteremia [61]. However, this result was not confirmed in a study with a low baseline catheter-related bloodstream infections rate [62]. A recent, multicenter, prospective study reported the efficacy of a new catheter lock solution combining 7% sodium citrate, 0.15% methylene blue, and paraben for preventing DC related bloodstream infections compared with heparin solution [63]. Concentrated ethanol instilled in the DC lumen for a short dwell time could be an attractive antimicrobial solution, because it acts against bacteria and fungi and is able to eradicate biofilm [64].

Very few data are available on the use of DC antimicrobial lock in critically ill patients. The effectiveness of 46.7% citrate locks versus saline locks, for delaying DC related infections, was recently suggested in a randomized control study involving 78 critically ill patients [20]. However, there was a very high incidence of DC related bloodstream infections in both the citrate (24/1,000 catheter days) and the saline (30/1,000 catheter days) groups. For instance, in a survey of 534 DCs inserted in 289 critically ill patients currently locked in the interdialytic period with 4% or 30% trisodium citrate, the rate of DC related bloodstream infections was 1.6/1,000 catheter days [19], and therefore whether the results of the study of Hermite and colleagues can be extrapolated to ICUs with lower rate of DC-related bacteremia is questionable [20]. Although some antimicrobial locks appear to be promising, further studies are necessary before the use of antimicrobial lock can be recommended for preventing and/or treating DC infections.

##### Guidewire exchange

DC replacement over guidewire exchange in patients with a suspicion of DC infection is a common practice in many ICUs. A systematic review including ICU patients with different type of catheters suggest that compared with new-site replacement, guidewire exchange is associated with fewer mechanical complications but with a trend toward a higher rate of catheter colonization, regardless of whether patients have a suspected infection [65]. In a recent study in critically ill pediatric patients, guidewire exchange was the only identified risk factor for catheter-related bloodstream infection [66]. Several studies suggest that catheter guidewire exchange in conjunction with systemic anti infectious treatment is an acceptable way to manage mild catheter-related infections in chronic hemodialysis patients. This strategy, called the salvage of site approach, has a low complication rate with no increase in bacteremia episodes and provides similar longevity to new-site placements [17,67-70]. In ICU patients with suspected DC infection, there is a lack of evidence to support or discourage such a strategy. It could be proposed as an alternative option to DC removal in hemodynamically stable patients without endocarditis, thrombosis, or other DC-related focus of infection. In our opinion, the new DC, which has been changed over guidewire, should be removed for a new site placement when shock develops, or in the event of persistent fever or persistent positive blood cultures. We cannot yet say whether the new DC, which has been changed over guidewire, should be removed if the initial DC tip culture yielded a positive result.

## Conclusions

Dialysis catheter management is a major factor in ICU renal replacement therapy. The type of catheter and catheterization procedures, especially the insertion site and catheter maintenance (flushes, locks), affects the quality of RRT and the risk of catheter dysfunction. In patients on intermittent hemodialysis, the right internal jugular insertion site should be preferred to deliver the best RRT dose, if the prescribed Q_B_ is higher than 200 ml/min. Hygiene procedures that are the same as for central venous catheters are of paramount importance to limit the risk of infection. Further studies are needed to determine the indication of antimicrobial locks, such as ethanol, to prevent DC infections. The insertion site does not influence catheter infection rate except for certain subpopulations. To prevent DC colonization, the physician has to be vigilant for the jugular position when BMI < 24 and the femoral position when BMI > 28. Practitioners should bear in mind that patients with acute kidney injury are likely to become chronic dialysis patients and that it is therefore essential to preserve ulterior potential vascular access. This is why the subclavian insertion site is not recommended. The training of teams managing patients with DC and the teaching procedures need to be evaluated.

### Key messages

Point 1 DCs should have an outer diameter of at least 12 Fr and a length of at least 15 cm in the jugular position to obtain right atrium placement for soft DC, superior vena cava for rigid DC, and probably at least 24 cm in length in the femoral site.

Point 2 The left jugular position is associated with a higher DC dysfunction rate. In patients on intermittent hemodialysis, the right internal jugular insertion is associated with a higher delivered dialysis dose if the prescribed *Q*_B_ is higher than 200 ml/min. To prevent DC colonization, the physician has to be vigilant for the jugular position when BMI < 24 and the femoral position when BMI > 28.

Point 3 Partial thrombosis of the catheter lumen, as evidenced by frequent pressure alarms, leads to a decrease in QB that reduces RRT dose. In the event of DC dysfunction, DC replacement over guidewire can be performed particularly if DC dysfunction results from a malpositioned DC.

Point 4 No evidence of the efficacy of antimicrobial lock and antimicrobial-coated DC in preventing DC infections.

Point 5 AKI is an independent risk factor for end-stage renal disease, and preserving ulterior potential vascular access is essential. This is why the subclavian insertion site is not recommended.

## Competing interests

The authors declare that they have no competing interests.

## Authors' contributions

JFT, BS, and AL made substantial contributions to the conception of the review. NM and BS drafted the manuscript. All authors critically revised the manuscript and approved the final version of the manuscript submitted for publication.
